# The mechanism of seepage prevention of polymer grouting repair for longitudinal cracks in reconstructed and expanded pavement

**DOI:** 10.1371/journal.pone.0332219

**Published:** 2025-10-06

**Authors:** Lin Guo, Baolin Wang, Bei Zhang, Zhaoxu Yang, Lin Zhou, Yanhui Zhong

**Affiliations:** 1 Henan Transport Investment Testing and Certification Co., Ltd., Zhengzhou, China; 2 Henan College of Transportation, Zhengzhou, China; 3 Henan Jiaoyuan Engineering Technology Group Co., Ltd, Zhengzhou, China; 4 School of Water Conservancy and Transportation, Zhengzhou University, Zhengzhou, China; 5 The Key Laboratory of Road and Traffic Engineering, Ministry of Education, Tongji University, Shanghai, China; 6 Henan Transport Investment Group Co., Ltd., Zhengzhou, China; Mirpur University of Science and Technology, PAKISTAN

## Abstract

A key challenge in highway reconstruction and expansion projects is to reduce longitudinal cracking at the junction of old and new roadbeds, which is a critical issue in the maintenance of transportation infrastructure. Polymer grouting technology, a non-excavation method for hidden subgrade defects, leverages material self-expansion properties to fill voids, enhance load-bearing capacity, and bond structural layers while providing seepage control—enabling rapid repair of longitudinal pavement joints. This study investigates the seepage characteristics of polymer-grouted longitudinal joints in reconstructed pavements by simulating post-repair infiltration behavior under rainfall conditions. The results elucidate the anti-seepage efficacy of polymer grouting, offering theoretical and practical insights for efficient longitudinal joint treatment in highway expansion projects.

## Introduction

After pavement construction, under the combined effects of repeated traffic loads and natural factors like rainfall, a series of distresses often emerge within just two or three years of service [1–2]. These include cracks, spalling, and loosening. In the case of widened or reconstructed pavements, such issues are particularly prone to occur at the joints between old and new pavement sections. When exposed to rainfall, these structural damages can significantly affect the pavement’s mechanical properties and seepage behavior [3–4]. Therefore, studying the seepage characteristics of longitudinal pavement joints is of great importance.

The study of seepage characteristics of pavements containing longitudinal joints has always been an important topic of concern in the field of traffic engineering and geotechnical engineering, especially in the areas of pavement drainage performance, stability of road base and environmental protection. Based on this, scholars at home and abroad have carried out investigations on the seepage mechanism, and in the middle of the 19th century, Darcy [5] In the mid-19th century, Darcy carried out experiments on seepage in sandy soil. He studied the flow law of water in porous media (such as sandy soil) through a series of rigorous experiments, and based on the results of the experiments, he proposed Darcy’s law which describes the relationship between the water permeability and hydraulic gradient under the conditions of laminar flow, and the result is of great significance in many fields, such as hydrology, soil mechanics and so on. Subsequently, Richards [6] Based on Darcy’s law, he carried out a large number of seepage experiments and proposed a control equation for unsaturated water movement, which is now widely used in the study of seepage characteristics of saturated-unsaturated soils.J. R. Philip [7–8] Extensive simulation studies based on the Richards equation have given solutions to the full nonlinear unsaturated flow equation for the infiltration problem on a plane hillslope of a homogeneous isotropic soil with uniform initial water content, with minor modifications and simplifications for two simple types of hillslope anisotropy.

Based on the development of seepage theory, scholars gradually realize that rainwater infiltration caused by road cracks is the key factor affecting road stability. In terms of research on disease development characteristics: Zou, Y. [9] taking cold region highways as the main research object, the development law of cracks on cold region highways under the influence of freezing and thawing was analyzed. Wang, H. [10] summarized the forms of diseases in the renovation and expansion of highways, and believed that longitudinal joints at junctions are the main diseases in the renovation and expansion of highways. Combined with the strength of road structures, he analyzed the causes of crack diseases. Chen, G. H. [11] conducted extensive research on engineering cases, analyzed the possible forms and characteristics of diseases that may occur in the widening of old roadbeds, and proposed corresponding control methods. Han, F. F. [12] taking the highway renovation and expansion project as an example, focuses on analyzing the causes of longitudinal joints in the road surface at the joint position of the renovated and expanded highway, and proposes a control plan during construction.

In terms of studying the impact of rainwater infiltration on roadbed stability, Ma, N. Z. [13] constructed a numerical analysis model for road reconstruction and expansion in mountainous and hilly areas, studied the influence of continuous rainfall on the seepage field of reconstructed and expanded roadbeds, and believed that continuous infiltration was the direct factor leading to instability of reconstructed and expanded roadbeds. Yue, J. W. [14] analyzed the strength changes of roadbed soil after rainwater infiltration and summarized the damage and failure laws of soil caused by water infiltration. The study focuses on the high fill roadbed of the underlying coal bearing strata in Lan, G. G. [15] with a particular emphasis on analyzing the disaster process caused by rainwater infiltration on the slope of the fill roadbed, and exploring the destructive law of rainwater on the stability of the fill roadbed slope.

From the above research, it can be seen that rainwater infiltration along road cracks can lead to roadbed instability. Therefore, based on the unsaturated seepage theory, this paper establishes two-dimensional numerical models of the reconstructed pavement under different working conditions with the help of finite element software, simulates the rainfall infiltration process before and after the repair of pavement cracks with high polymer grouting, researches the change rule of the internal pore water pressure, volumetric water content and transient saturated zone of the embankment with the time of rainfall, and compares and analyses the seepage characteristics of the embankment under different working conditions, so as to reveal the anti-seepage mechanism of longitudinal joints of the pavements reconstructed by high polymer grouting. The seepage control mechanism and seepage control effect of longitudinal joints of road surface.

## 1. Saturated-unsaturated seepage principle

When studying the seepage of embankment slopes under rainfall conditions, special attention needs to be paid to the interaction of groundwater flow in the saturated and unsaturated zones within the slope. During rainfall, rainwater will gradually infiltrate into the soil, and part of the soil close to the ground surface may reach saturation quickly due to rainfall, while with the increase of depth, the water in the soil pores gradually decreases, forming a non-saturated zone, which is therefore known as the saturated-unsaturated seepage problem [16]. Saturated-unsaturated seepage still follows Darcy’s law of non-constant seepage within the soil, and the partial differential equation takes the form of equation (1) as follows [17]:


$$\frac{\partial }{\partial x}\left(k_{x}\frac{\partial h}{\partial x}\right)+\frac{\partial }{\partial y}\left(k_{y}\frac{\partial h}{\partial y}\right)+w=m_{w}\rho_{w}g\frac{\partial h}{\partial t}$$ (1)

Where: *h* is the total head height in the rock fissure; and *k*_*x, ky*_ is the permeability coefficient in the x and y directions; *w* is the source-sink term; *m*_*w*_ is the specific water capacity; *ρ*_*w*_ is the water density; *g* is the acceleration due to gravity; and *t* is time.

For unsaturated seepage calculations, the permeability coefficient of a geotechnical body is closely related to the volumetric water content of the saturated soil and the matrix suction, and a soil-water characteristic curve is commonly used to describe this relationship. Although Darcy’s law is still applicable when dealing with problems related to unsaturated soils, its permeability coefficient is no longer a fixed value, but is closely related to the matrix suction of the soil or the volumetric water content of the soil body [18]. Prediction methods for soil-water characteristic curves rely heavily on experimental point data, to which existing models are fitted. A common method used in the past to determine the infiltration coefficient was to use the classical Van Genuchten model [19]. Numerical fitting was carried out with the model expression:


$$\theta \;=\frac{\theta_{s}-\theta_{r}}{{\left[1+{\left|\alpha h\right|}^{n}\right]}^{m}}+\theta_{r}\left(m=1-\frac{\text{1}}{n},\;0<m<1\right)$$ (2)

$$\;K\left(\theta \right)=K_{s}{\left(\frac{\theta -\theta_{r}}{\theta_{s}-\theta_{r}}\right)}^{\frac{\text{1}}{\text{2}}}{\left\{1-{\left[1-{\left(\frac{\theta -\theta_{r}}{\theta_{s}-\theta_{r}}\right)}^{\frac{\text{1}}{m}}\right]}^{m}\right\}}^{\text{2}}$$ (3)

Where: *θ* is volumetric water content; *θ*_*s*_ is residual water content; *θ*_*r*_ is saturated water content; *K*_*s*_ is saturated permeability coefficient; *m*, *n* are shape parameters of soil-water characteristic curve.

## 2. Tests on the impermeability characteristics of polymer materials

According to relevant literature [20–21] According to the literature, the self-consolidating skin formed by the polyurethane polymer grouting material after construction can effectively isolate the intrusion of external water, which greatly enhances the waterproof performance of the material. At the same time, its internal structure is also very unique. Up to 95% of the closed pores are in the form of high-strength interconnected walls, and it is because of this special closed pore structure that the polyurethane polymer grouting material shows excellent impermeability, with a permeability coefficient as low as 10^−8^ cm/s. Therefore, in order to more intuitively explore and study the permeability characteristics of the polymer material, this section carries out the test of the permeability resistance of the polymer material.

### 2.1 Test methods

High polymer material is a kind of rigid polyurethane foam, so this section adopts the test method of permeability in JC/T 998–2006 Spray Polyurethane Rigid Foam Insulation Material. According to the standard, the specimen size of the permeability test is 100 × 100 × 30 mm, and three specimens are tested as a group and the value is taken as the test result. Since this method is similar to the method and principle of the variable head permeability test, this section adopts the formula of the variable head permeability test to calculate the permeability coefficient of the polymer material:


$$K=\frac{a\cdot L}{A\cdot t}ln\left(\frac{h_{\text{1}}}{h_{\text{2}}}\right)$$ (4)

Where: is the permeability coefficient (cm/s); is the cross-sectional area of the test tube (cm^2^); is the height of the test block cm) is the cross-sectional area of the test block cm); is the test time (s); is the head value at the beginning cm); is the head value at the end (cm).

### 2.2 Experimental process

**Fig 1:**
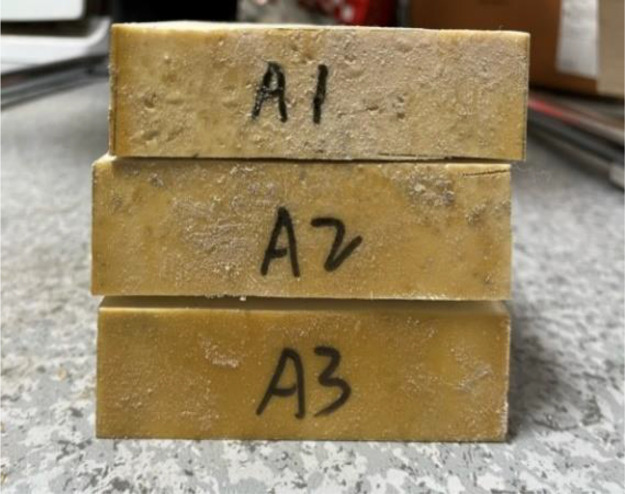
Preparation of specimens.

(1) Preparation of specimens: the polymer specimens obtained by grouting in a steel mould of 100 × 100 × 100 mm will be cut into the dimensions required for the permeability test, i.e., 100 × 100 × 30 mm, with the same density of three specimens in each group (see Fig 1), and four densities, i.e., four groups, are set up, respectively, as 0.32 g/cm3, 0.41 g/cm3, 0.50 g/cm3, 0.59 g/cm3;
(2) Place the prepared specimen horizontally and place a glass tube with a diameter of 20 mm and a length of 1100 mm vertically on its upper surface, mark the height of the glass tube at 1000 mm, and seal the gap between the glass tube and the specimen with a n

### 2.3 Impermeability properties of polymers at different densities

**Fig 2:**
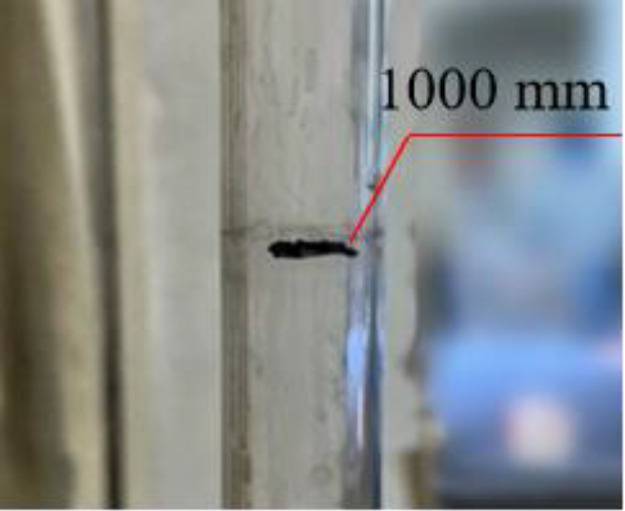
Marking heights.

**Fig 3:**
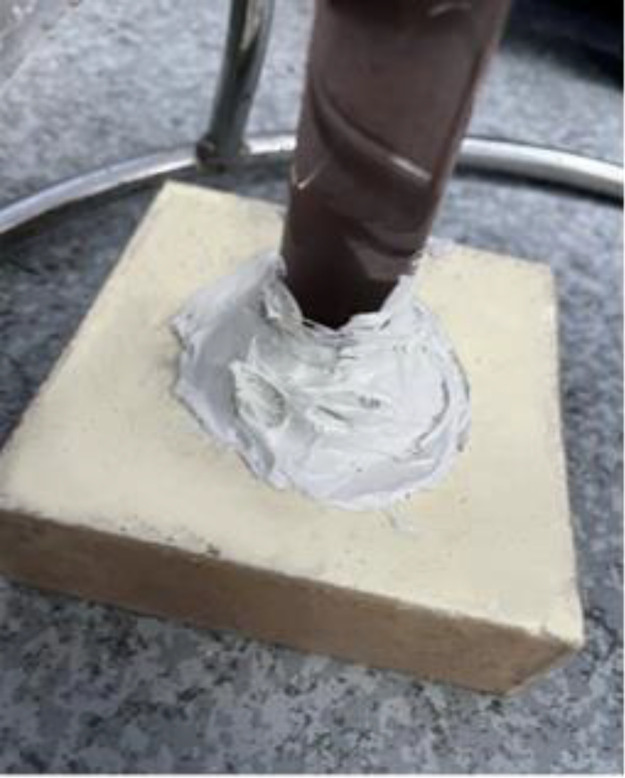
Sealing the gap.

**Fig 4:**
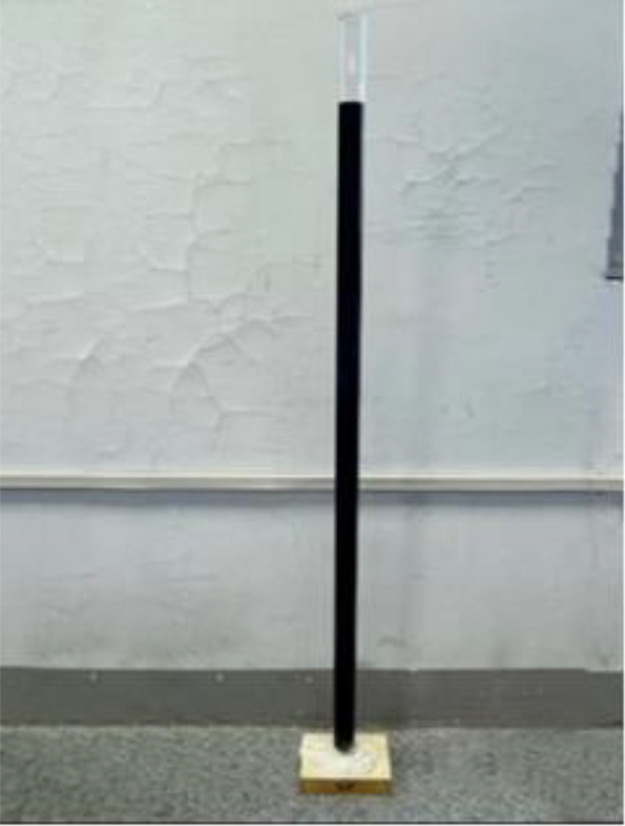
Adding the solution.

**Fig 5:**
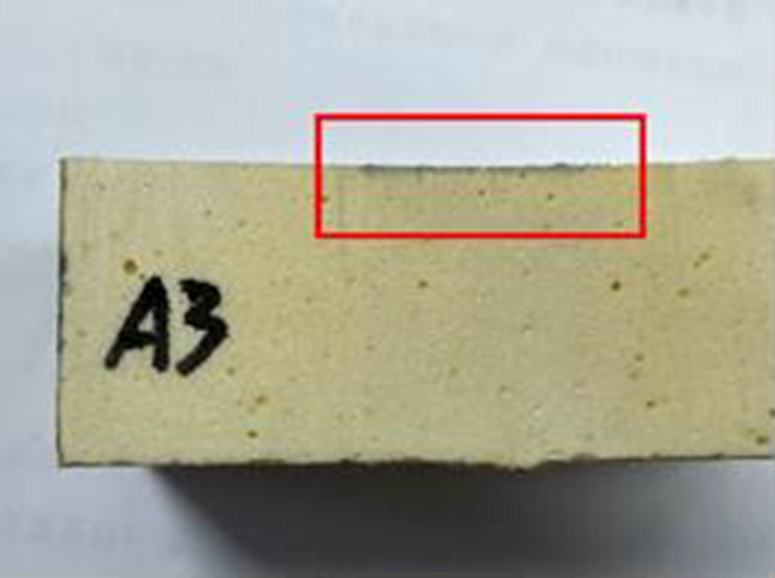
Density of 0.32 g/cm^3^.

**Fig 6:**
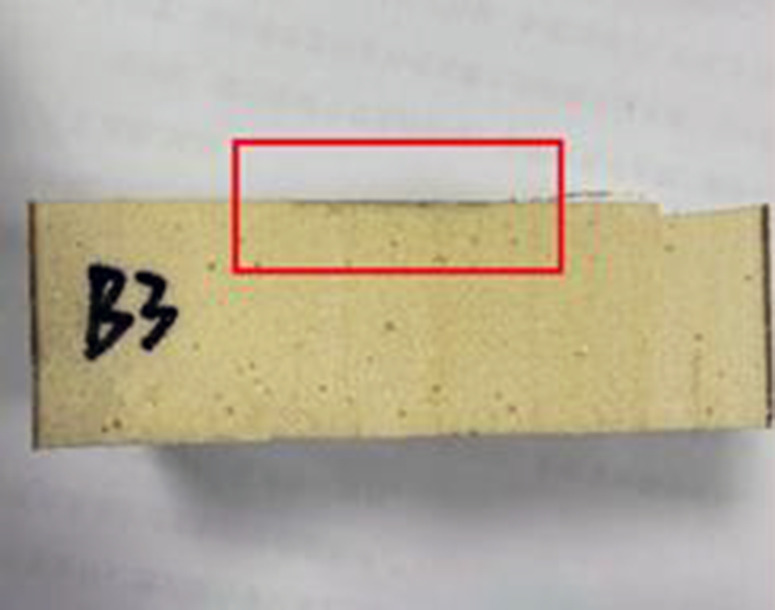
Density of 0.41 g/cm^3^.

**Fig 7:**
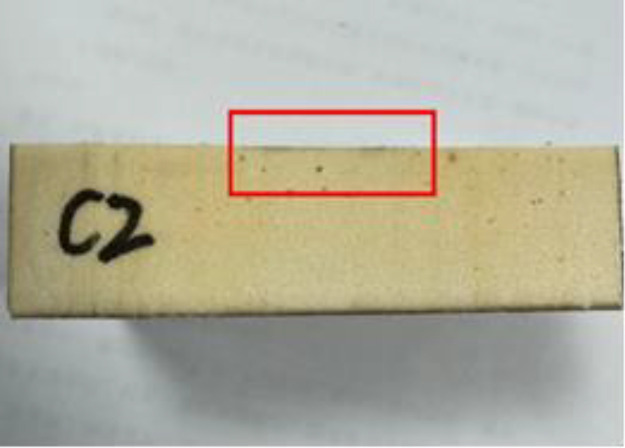
Density of 0.50 g/cm^3^.

**Fig 8:**
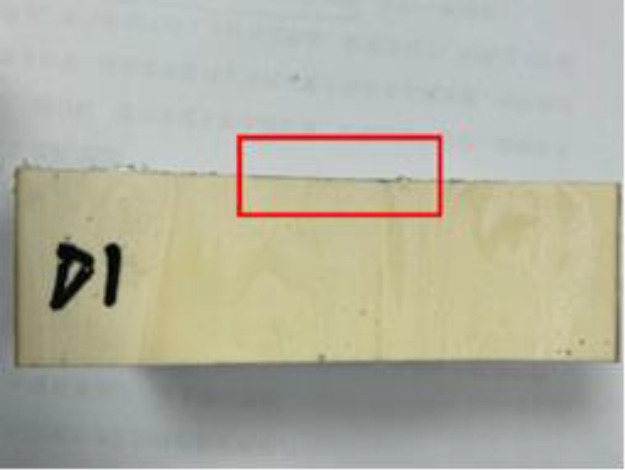
Density of 0.59 g/cm^3^.

After cutting the specimen, comparing the degree of solution infiltration at different densities, it can be seen that: the smaller the density of the specimen, the deeper the infiltration; as the density increases, it can be seen that the interior of the specimen is more dense and the porosity of the material is smaller, making it difficult for aqueous solutions to infiltrate, thus indicating that the high-density material has a better resistance to infiltration. The following figure shows the more obvious results in each group (see Figs 2–8).

**Fig 9:**
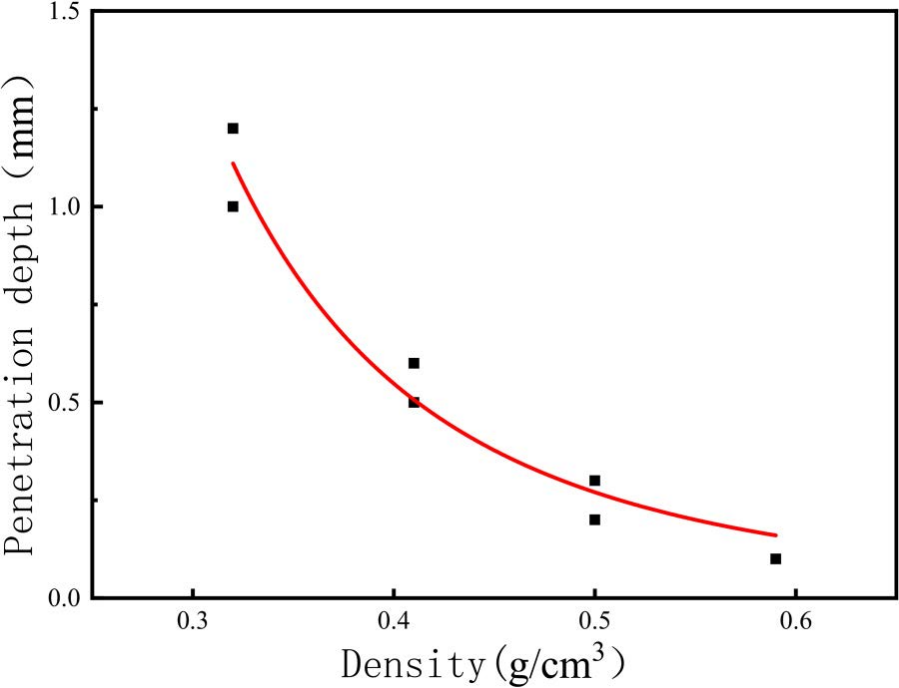
Fitted depth of infiltration of aqueous solutions in polymers at different densities.

All the test results are summarised in Table 1 below. According to Fig 9. It can be seen more intuitively: with the increase in density, the specimen penetration depth gradually decreases. For the density of 0.59 g/cm^3^ specimen, the penetration depth is only 0.1 mm, which is almost negligible. According to the trend of the curve it can be seen that when the density increases to a certain value, the depth of the material penetration tends to be 0; with the increase in density, the permeability coefficient of the specimen also decreases. density increases, the permeability coefficient of the specimen also decreases. The density of the commonly used polymer material is 0.3 g/cm3, and the corresponding permeability coefficient is only 1 × 10^−8^ cm/s, which is consistent with the existing conclusion.

**Table 1:** Test results of permeability of polymers with different densities.

grade	Density (g/cm^3^)	Depth of penetration (mm)	Median (mm)	Permeability coefficient (cm/s)
A1	0.32	1	1	1 × 10^−8^
A2		1.2		
A3		1		
B1	0.41	0.5	0.5	0.5 × 10^−8^
B2		0.5		
B3		0.6		
C1	0.50	0.3	0.3	0.3 × 10^−8^
C2		0.2		
C3		0.3		
D1	0.59	0.1	0.1	0.1 × 10^−8^
D2		0.1		
D3		0.1		

The fitted equation for the depth of penetration of an aqueous solution in a polymer as a function of density is shown in equation (5) below:


$$\begin{array}{c} \{\begin{array}{c} {}*20l\;\;\;\;\;\;y=0\mathrm{.03017}x^{-3\mathrm{.16446}}\\ {\;\;\;R}^{\text{2}}=0\mathrm{.95469} \end{array} \end{array}$$ (5)

Through the polymer material water absorption test and impermeability test can again confirm the foaming non-water reaction two-component polyurethane material is a kind of impermeable material, the internal material is not easy to absorb water and has good impermeability performance, but also for the following under the rainfall conditions of polymer grouting repair longitudinal joints in the pavement to provide a basis for the research.

## 3. Finite element modelling

In order to study the changes of slope seepage field under different rainfall events and pavement conditions, an artificial slope model is set up for the reconstructed and expanded asphalt pavement structure in this section. In order to better analyse the repair effect of polymer grouting, the finite element model is set up with three working conditions, namely, the reconstructed pavement, the cracked pavement, and the repaired pavement using polymer grouting.

### 3.1 Model dimensions

**Fig 10:**
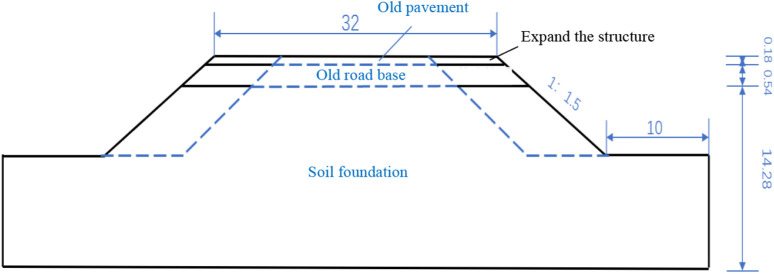
Slope model of the reconstructed pavement (unit: m).

In order to consider the change of seepage field of the pavement under rainfall conditions, the road base structure was simplified appropriately (see Fig 10), which consists of 18 cm asphalt concrete surface layer, 54 cm cement stabilised crushed stone base layer, and 14.28 m soil base from the top to the bottom. The total width of the reconstructed pavement is 32 m. The slope ratio of the side slopes is 1:1.5, and the foot of the slope extends outward for 10 m.

**Fig 11:**
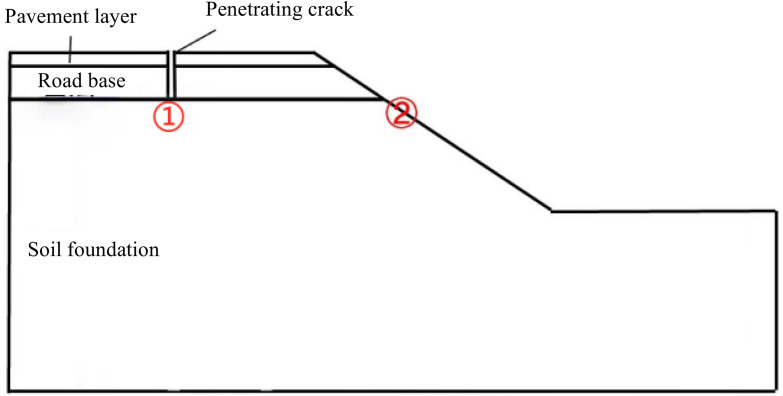
Longitudinal seam and monitoring section setup.

For the pavement with longitudinal joint condition and grouting repair condition, the longitudinal joint and grouting position are set at the junction of old and new surface layer, the depth is through the surface layer and the base layer, and the width is 2 cm. In order to facilitate the analysis and observation of the change rule of seepage characteristics at different positions, two monitoring points are selected for analysis in this section, namely: the top of the soil base under the longitudinal joint ①, and the monitoring point at the right edge of the soil base ②. Considering the symmetrical structure of the pavement, 1/2 model is taken for numerical calculation and analysis, as shown in Fig 11.

**Fig 12:**
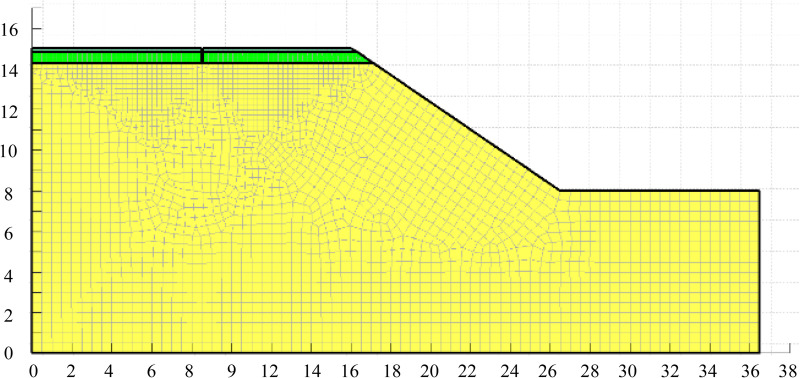
Model meshing.

Fig 12 shows the calculation model under the condition of containing longitudinal joints. The model mesh division adopts quadrilateral & triangular cells, the mesh size is 0.5 m, the global total of 2612 nodes, 2502 cells. The cell setting and meshing of the model for the other two conditions are the same as above.

### 3.2 Parameter selection

Using the indoor test results in Chapter 2, combined with the structural material parameters of previous experimental studies [22], the corresponding parameter values were selected according to the needs of the thesis analysis. The values of the parameters of each structural layer of the model are shown in Table 2.

**Table 2:** Selection of parameters for road base pavement materials.

Makings	Permeability coefficient (m/s)	Saturated moisture content	Residual moisture content
Bituminous concrete surface	1.5 × 10^−6^	0.08	0.01
Cement stabilised aggregates	1.22 × 10^−6^	0.15	0.065
Soil foundation	3.3 × 10^−7^	0.2	0.053
polymer	1 × 10^−10^	–	–

**Fig 13:**
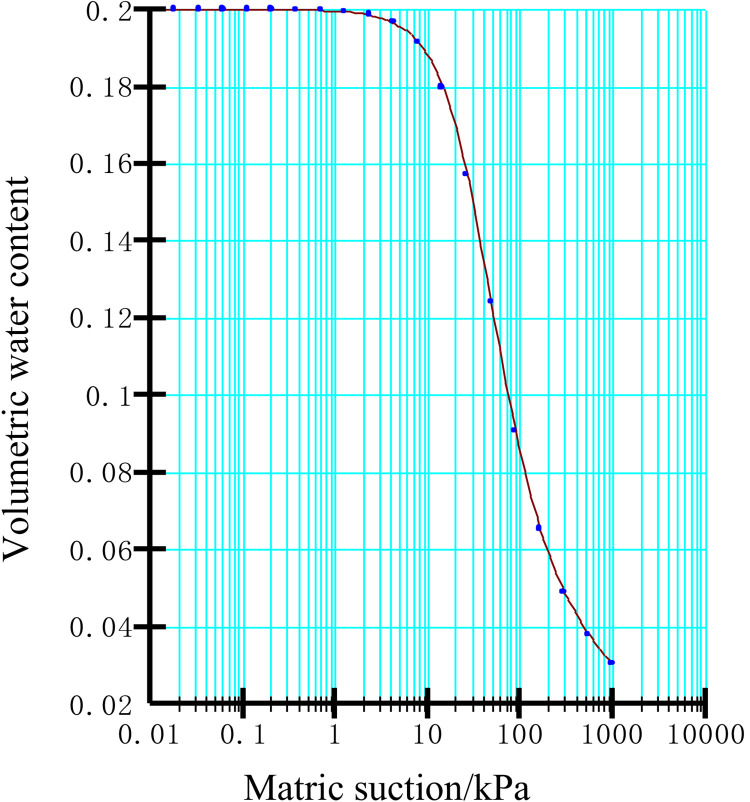
Soil-water characteristic curve.

**Fig 14:**
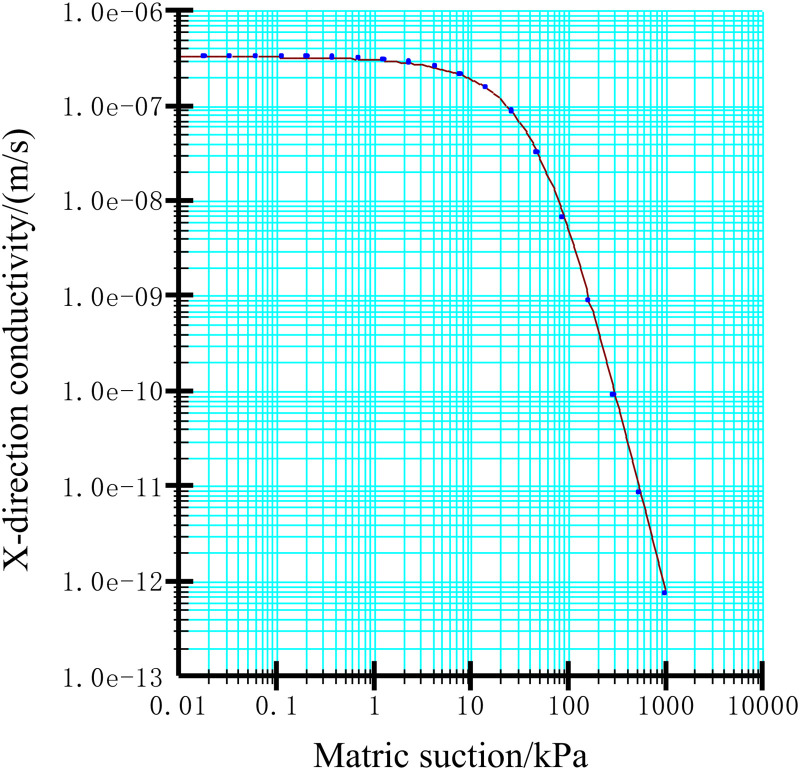
Penetration function curve.

Using the built-in sample function of the software, the soil-water characteristic curve of the residual slope accumulation can be simulated and fitted based on the measured data or known soil physical property parameters. Next, based on the fitted soil-water characteristic curve, the Van Genuchten model can be applied to further derive the infiltration function curve of the unsaturated region inside the soil body, as shown in Fig 13 and 14 From the following figures, it can be clearly seen that there is a significant non-linear connection between the matrix suction of the soil body and its volumetric water content and permeability coefficient.

### 3.3 Boundary conditions

The bottom and two sides of the model are set as impermeable boundaries, and the unit flow boundary transformed from the rainfall intensity boundary is imposed on the model surface. According to the rainfall class classification in China, this paper selects the daily average rainfall of 30 mm/d under heavy rainfall class as the flow boundary imposed on the slope surface, and the rainfall lasts for 3 days. The initial pore water pressure distribution in the calculated steady-state seepage field is used as the starting condition for the next transient analysis (Table 3).

**Table 3:** Classification of rainfall in the country.

Degree of rainfall	Drizzle	Rainy	Heavy rain	Storms	Torrential rain	Extremely heavy rainstorm
Daily rainfall (mm)	<10	10 ~ 25	25 ~ 50	50 ~ 100	100 ~ 250	>250

For the calculation case of pavements containing longitudinal joints, the perimeter of the crack is set as a constant head boundary, and the infiltration rate at the crack is based on the surface water infiltration rate test results of the old pavement, using the requirements of the Highway Drainage Design Code [23]. Since cracks are the main pathway for water infiltration into the interior of the pavement structure, the theory proposed by Ridgeway et al. advocates using the infiltration rate of cracks or joints as a measure [24] The design infiltration rate of surface water for asphalt pavements where cracks exist at the location of the surface layer is specified as 0.625 cm^3^/h/cm2, and the infiltration is calculated based on the number of cracks according to the following equation (6):


$$Q_{i}=I_{a}B+k_{P}B$$ (6)

Where: *I*_*a*_ is the design infiltration rate of surface water per square metre of cracked asphalt pavement (m^3^/d/m^2^); *k*_*p*_ is the infiltration rate of surface water to the surface of uncracked pavement per square metre.

## 4. Characterisation of seepage in longitudinal joints of reconstructed pavements under rainfall conditions

### 4.1 Pore water pressure

**Fig 15:**
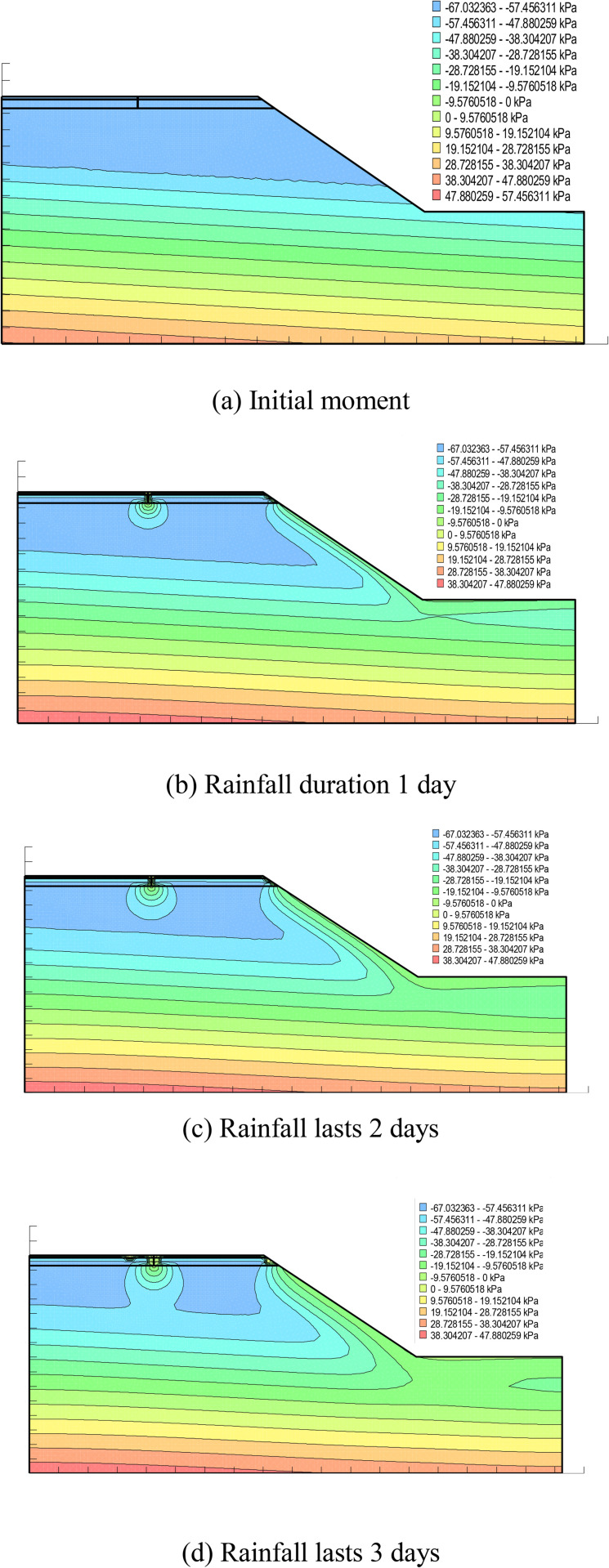
Pore water pressure distribution for different rainfall durations. (a) Initial moment. (b) Rainfall duration 1 day. (c) Rainfall lasts 2 days. (d) Rainfall lasts 3 days.

From the above Fig 15 can be seen: the existence of cracks in the pavement state, rainwater will be along the cracks penetrate into the pavement inside, with the increase of rainfall time, the pore water pressure at the cracks is a droplet-like slowly downward diffusion, in the third day and the pore water pressure contour within the soil base through. In the initial state, the pore water pressure inside the roadbed decreases gradually from top to bottom, and the pore water pressure in the upper part is negative, i.e., unsaturated state, and rainwater is more easily infiltrated, while it is positive in the middle and lower parts of the soil base, indicating that the middle and lower layers are in a saturated state and there is a negative substrate suction force. The longer the rainfall time, the more water enters into the roadbed, and the greater the increase in pore water pressure. As can be seen from the above figure, the pore water pressure at the top of the soil base in the initial state is in the range of −67 kPa ~ −57 kPa, the pore water pressure rises to the range of −9.6 kPa ~ 0 kPa during 1 day of rainfall, and the maximum pore water pressure at the top of the soil base reaches 0 kPa ~ 9.6 kPa after 2 and 3 days of rainfall, which indicates that the existence of cracks enables rainwater to infiltrate into the interior of the soil base directly, and the pore water pressure at the top of the soil base is affected by rainfall. The pore water pressure at the top of the soil base is greatly affected by rainfall, and the pore water pressure at the top of the soil base has reached a positive value on the 2nd day of rainfall, indicating that the place is already in a saturated state at this time, and there exists a negative matrix suction.

**Fig 16:**
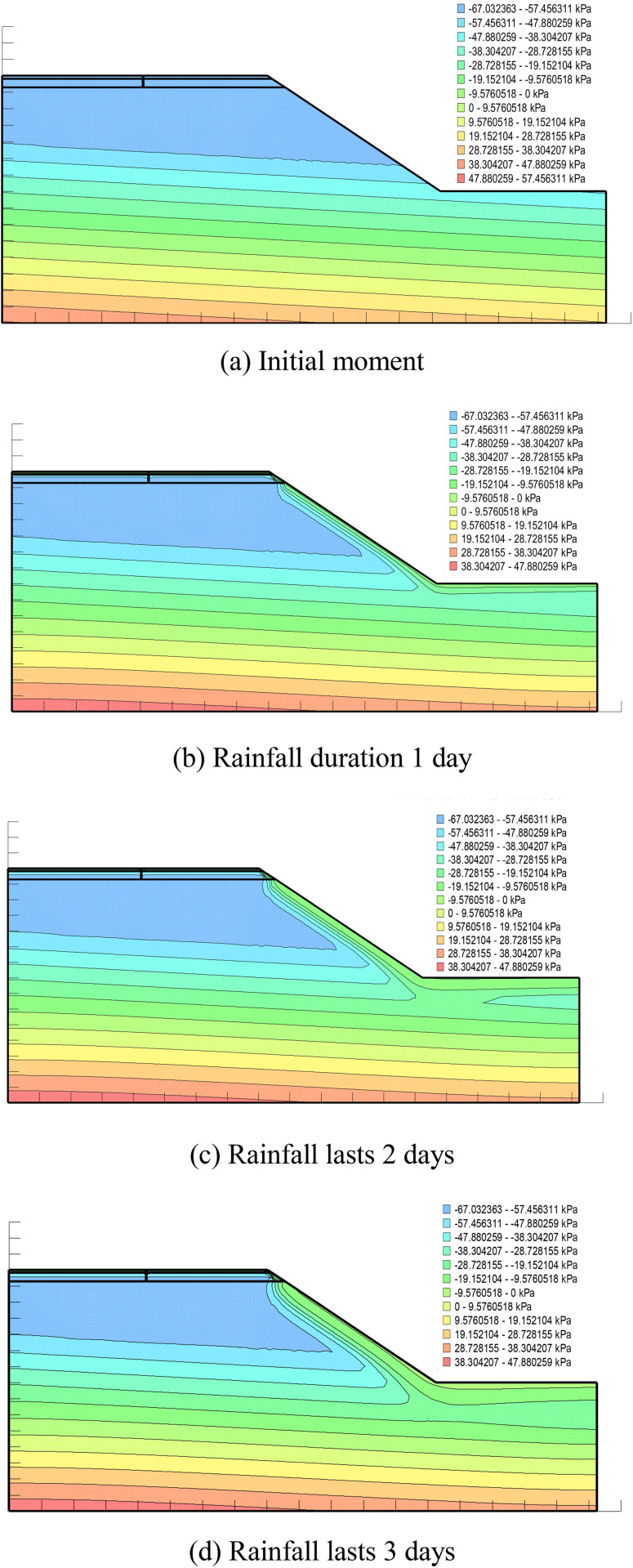
Pore water pressure distribution in grouted pavements under different rainfall durations. (a) Initial moment. (b) Rainfall duration 1 day. (c) Rainfall lasts 2 days. (d) Rainfall lasts 3 days.

From Fig 16, it can be seen that the pore water pressure inside the pavement at the initial moment gradually decreases from bottom to top, and with the increase of rainfall time, the rainwater continues to infiltrate and the pore water pressure contour keeps moving upwards, in which the pore water pressure value at the side slopes rises to a greater extent, and the change is more obvious compared with the pore water pressure at the grouting place.

**Fig 17:**
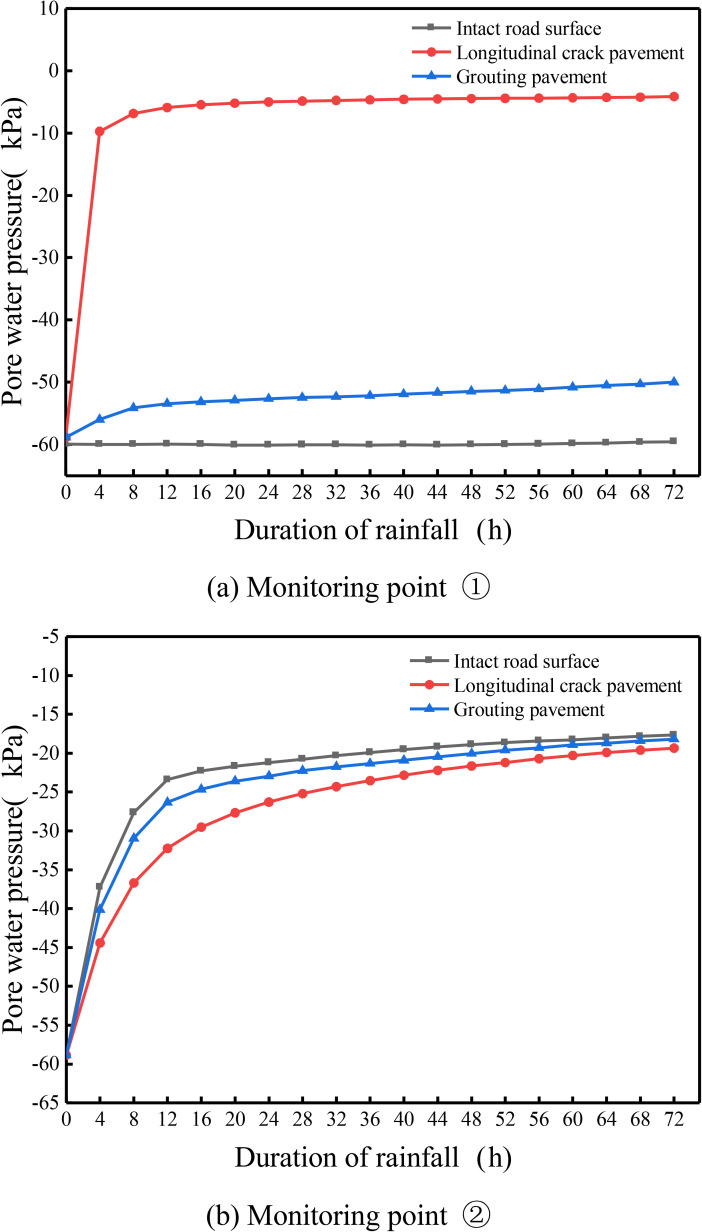
Variation of pore water pressure with time at each monitoring point under different working conditions. (a) Monitoring point ①. (b) Monitoring point ②.

According to the above analysis, it can be found that the pore water pressure inside the soil base is mainly in the longitudinal joints and slopes, which are more obviously affected by rainfall, so in order to more intuitively analyse the repair effect of the longitudinal joints of the high polymer grouted pavements, the longitudinal joints of the pavements, grouted pavements and intact pavements under the simulation results of the monitoring points ①② are compared and analysed, as shown in the following Fig 17.

From Fig 17 (a) above, the changes of pore water pressure with rainfall time at monitoring point ① under different working conditions can be seen: in the pavement intact condition, the pore water pressure at the point almost does not change with the rainfall time, and the whole is at the level of −60 kPa; for the longitudinal joints containing pavements, the pore water pressure at the point shows a trend of rapid increase and then smooth and unchanged, and with the increase in rainfall time, the maximum pore water pressure reaches −4.1 kPa. Reached −4.1 kPa, indicating that the point is close to saturation; longitudinal joints of the pavement by polymer grouting, the pore water pressure at the point increases slowly with the increase in rainfall time, and the longitudinal joints can be seen in comparison with the overall pore water pressure decreased significantly, and close to the intact condition, at the end of the rainfall when the pore water pressure reaches a maximum of −50.3 kPa, and the longitudinal joints of pavement with the maximum compared to the decline of 46.2 kPa, thus the pore water pressure at the point shows a trend of rapid increase and then smooth unchanged. 46.2 kPa. It can be seen that the pore water pressure at the longitudinal joint position after polymer grouting is greatly reduced, which makes the matrix suction inside the soil base increase greatly, so that the stability of the pavement can be improved.

From Fig 17(b) above, it can be seen that the pore water pressure at monitoring point ② under different conditions changes with rainfall time: the trend of pore water pressure at this point under the three conditions is generally similar, and it increases with the increase of rainfall time. Comparing the pore water pressure under the three conditions, it can be seen that the pore pressure of the intact pavement is larger than that of the grouted pavement and larger than that of the pavement with longitudinal joints, and the pore water pressure under the three conditions reaches −17.7 kPa, −18.2 kPa and −19.3 kPa respectively after the rainfall lasts for 3 days, which is caused by the fact that the monitoring point is located in the side slope position, and the pore water pressure of this point is affected by the level of the pore water pressure of the intact pavement in the rainfall condition. Under the rainfall condition, for the intact pavement, the point is subject to the double role of rainwater runoff and vertical infiltration of rainfall, while for the longitudinal joint pavement and grouted pavement, part of the pavement rainwater will be infiltrated along the longitudinal joints to the soil base, therefore, the amount of infiltrated rainwater at the side slopes will be reduced, which will make the pore water pressure at the point under the intact condition the largest value.

In summary, after the longitudinal joint pavement is repaired by using polymer grouting technology, the pore water pressure at different locations of the pavement under rainfall conditions is reduced to a certain extent, and the effect is most obvious at the longitudinal joints, which can improve the speed of the reduction of matrix suction of the pavement under rainfall conditions, and thus slow down the phenomenon of pavement collapse and slope landslides. Generally speaking, the pore water pressure value after grouting repair is relatively close to that of the intact pavement, indicating that the seepage characteristics based on pore water pressure after polymer grouting repair are improved to a great extent compared with that before grouting, although the same effect as that of the intact pavement cannot be achieved completely.

### 4.2 Pore water pressure

**Fig 18:**
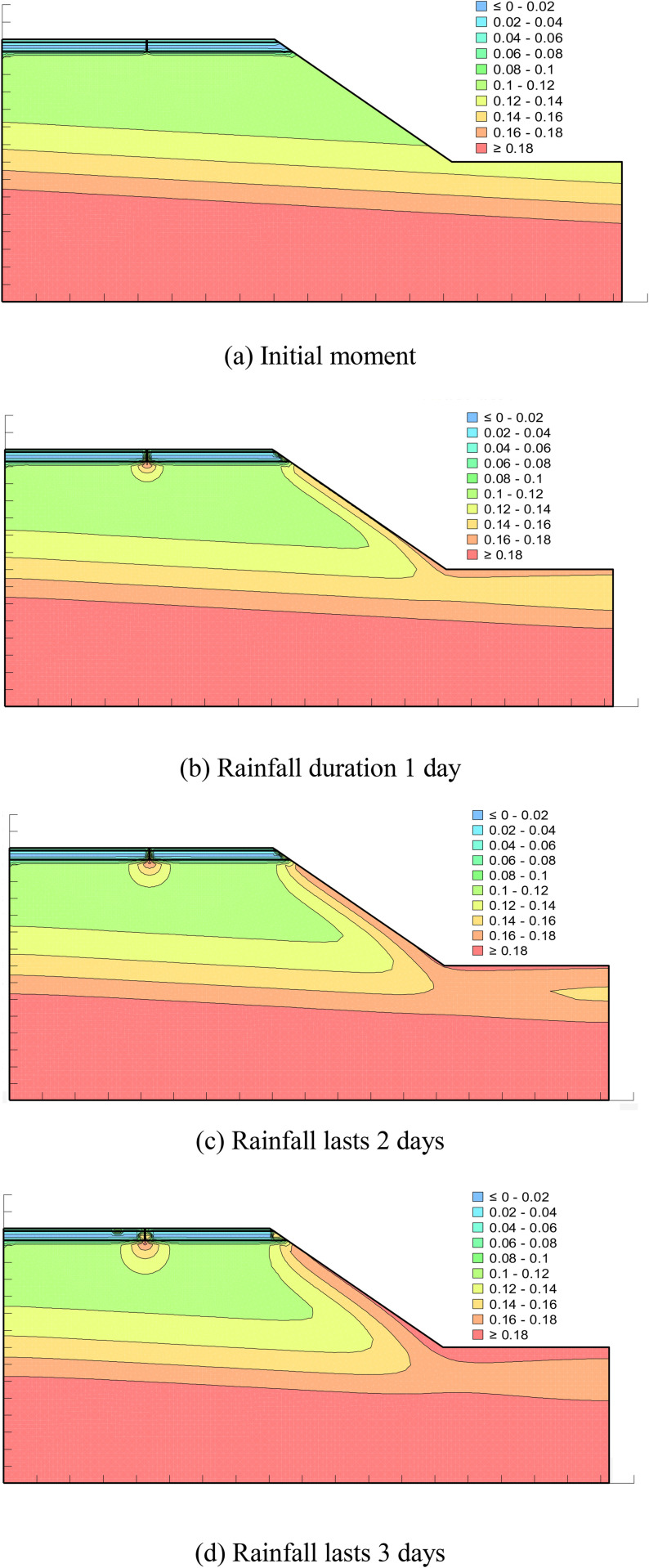
Distribution of volumetric water content for different rainfall durations. (a) Initial moment. (b) Rainfall duration 1 day. (c) Rainfall lasts 2 days. (d) Rainfall lasts 3 days.

From Fig 18 above, it can be seen that with the increase of rainfall time, the volumetric water content at the longitudinal joints and slopes changes the fastest and reaches the saturation state first. Rainfall, rainfall along the road to the longitudinal joints and slopes at the seepage, coupled with the slope by the direct infiltration of rainfall caused by the slope at the volumetric water content changes, on the other hand, with the continuous infiltration of rainfall, longitudinal joints at the volumetric water content in the form of raindrops continue to diffuse downward, so that the volumetric water content around the longitudinal joints is also increasing, and in a relatively short period of time to reach the state of saturation, which can be seen that the longitudinal joints have a great influence on the change of volumetric water content of the road surface. This shows that the existence of longitudinal joints has a great influence on the change of volumetric water content of pavement.

**Fig 19:**
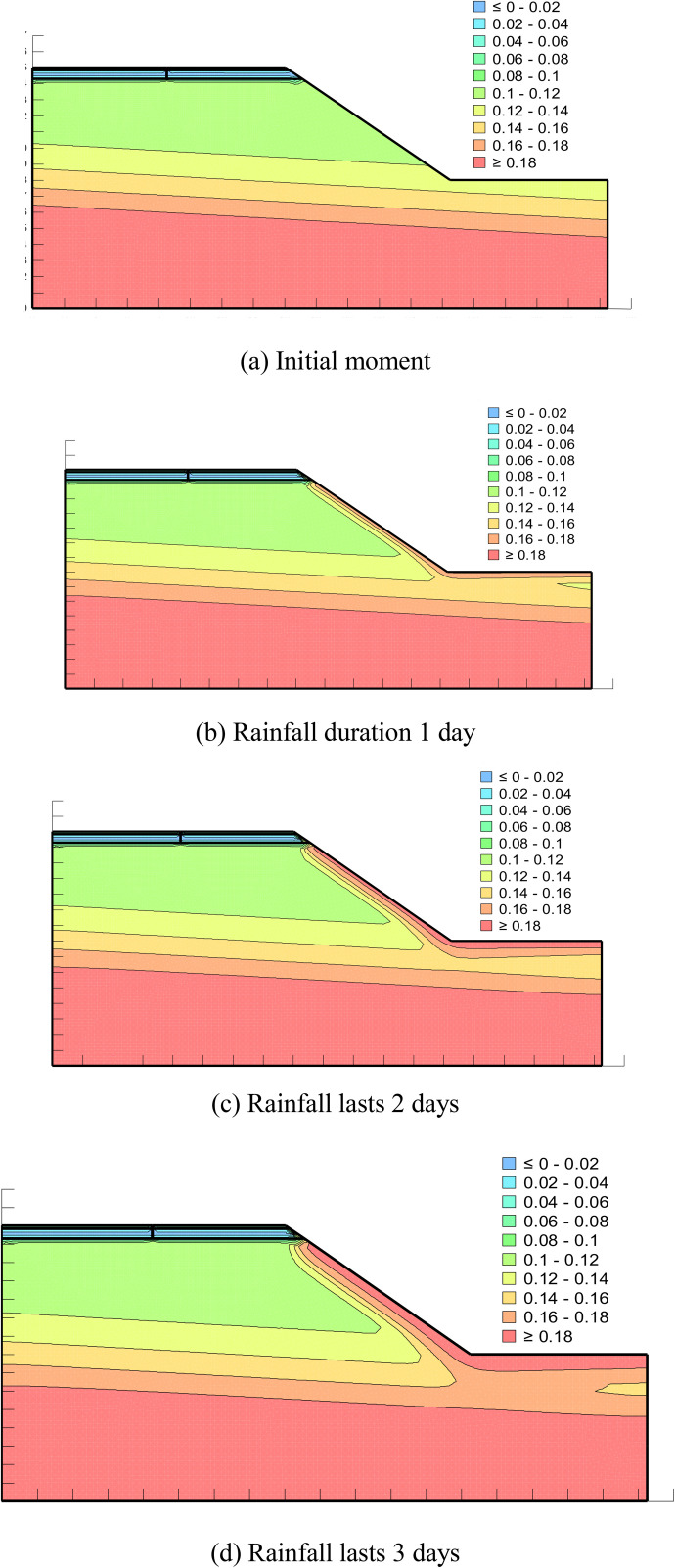
Distribution of volumetric water content of grouted repaired pavements under different rainfall durations. (a) Initial moment. (b) Rainfall duration 1 day. (c) Rainfall lasts 2 days. (d) Rainfall lasts 3 days.

From Fig 19 above, it can be seen that the initial moment of volumetric water content in the soil base structure layer presents from the bottom to the top the change rule of gradual decrease. This phenomenon is due to the soil base in the long-term rainfall conditions at the bottom of the continuous accumulation of water, resulting in the lowest layer of the volumetric water content tending to be saturated, and the upper layer of the soil due to the gravity of the rainwater continues to infiltrate the lower layer of the volumetric water content and the lower layer of the smaller compared to the. With the increase of rainfall time, it can be seen that the volumetric water content at the slope changes are more obvious. The larger the rainfall volume at the slope, the larger the volumetric water content. On the second day of rainfall at the surface of the slope is close to the saturated water content, and with the continuous infiltration of rainfall at the slope, the soil base is also slowly affected by the influence of the slope. The volumetric water content increases, the contour line continues to move upward. After grouting the longitudinal joints of the pavement, it is difficult for rainwater to infiltrate through the surface of the road, and the excess rainwater that is difficult to be discharged from the pavement will also flow to the slope, so that the volumetric water content at the longitudinal joints is almost insignificantly changed with the amount of rainfall, and only small changes occur between the contact surfaces of the structural layers.

**Fig 20:**
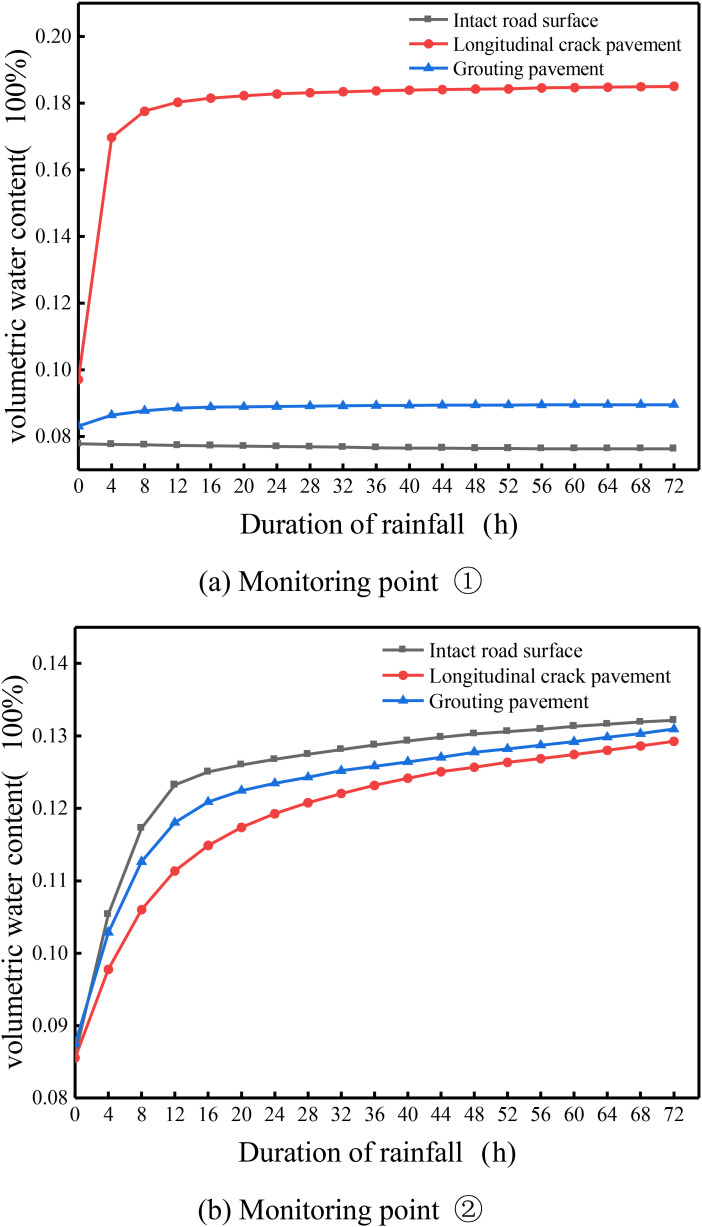
Variation of volumetric water content with time at each monitoring point under different working conditions. (a) Monitoring point ①. (b) Monitoring point ②.

From Fig 20 (a), the change of volumetric water content with rainfall time at monitoring point ① under different working conditions, it can be seen that: the volumetric water content at this point almost does not change with rainfall time under the condition of intact pavement, and the whole is at the level of 0.076; when there is a longitudinal joint in the pavement, the volumetric water content at this point changes a lot, and it shows that with the increase of rainfall time the volumetric water content at this point increases rapidly firstly and then stably, and the trend of no change is stable, and rainfall lasts until day 3, when this point reaches the maximum value of 0.185. When rainfall continues for the 3rd day, the volumetric water content at the point reaches a maximum value of 0.185, compared with the intact pavement increased by 0.109, and is close to the saturated water content. At this time the location of the soil body tends to be saturated. Pavement longitudinal joints by high polymer grouting after the point of the volumetric water content showed a slow increase in the trend, and the whole and the intact pavement is close to the end of the rainfall the point of the volumetric water content reached a maximum value of 0.09, compared with the longitudinal joints containing the pavement maximum reduced by 0.095, which can be seen in the longitudinal joints after the high polymer grouting the volumetric water content at the location of the location of a large degree of reduction in the soil base is in a non-saturated state, pavement The stability of the pavement is relatively improved.

From the above Fig 20 (b) different conditions of the monitoring point ② at the volumetric water content with rainfall time changes can be seen: the point in the three conditions of the volumetric water content change trend is similar, with the increase in rainfall time increases continuously, and the intact condition of the volumetric water content of the whole is greater than the grouted pavement is greater than the longitudinal joints in the pavement. In the rainfall of the three conditions of the maximum volumetric water content of 3 days, respectively, was 0.132. 0.131, 0.129, The reason for this phenomenon is that the monitoring point is located at the slope, which is subject to both runoff and vertical infiltration of rainfall for the intact pavement under rainfall conditions, whereas part of the rainfall on the road surface will infiltrate along the longitudinal joints or high polymer grouted joints to the interior under the other two conditions, which reduces the amount of rainfall infiltration at the monitoring point compared with the intact pavement. This reduces the infiltration of rainwater at this monitoring point compared to the intact pavement, resulting in a smaller volumetric moisture content than the intact pavement.

Through the above various analyses on the volumetric water content after grouting and repairing pavement longitudinal joints under rainfall conditions, it can be concluded that: the seepage characteristics of the pavement based on the volumetric water content after polymer grouting and repairing pavements have a certain gap with the intact pavements and can not reach the same level as that of the intact pavements, but there is a great degree of improvement compared to the longitudinal joints pavements, which has the most significant effect on the longitudinal joints. The maximum volumetric water content is reduced by 0.095% compared to that of the longitudinal joints pavement. Compared with the longitudinal joint pavement, the maximum volumetric water content is reduced by 0.095, and this kind of repair effect can slow down the disappearance of matrix suction within the soil body to a certain extent, avoiding the occurrence of various pavement diseases or slope disasters due to the saturation of volumetric water content.

### 4.3 Transient saturated zone and water table

Under the action of rainfall or drainage rain mist, the surface of the slope is the first to appear a certain area of saturated area, which is the transient saturated zone [25] The area is the transient saturated zone. Under the influence of rainfall, the saturation of the soil body increases with the infiltration of rainwater, while the transient saturated zone area also becomes larger, and produces a certain amount of transient water pressure. With the continuous growth of the saturated zone range, the depth and breadth of its influence increases synchronously, according to literature [26] According to the literature, the expansion of the transient saturated zone inside the slope will greatly affect the mechanical equilibrium state of the slope, and once it exceeds the carrying capacity of the slope itself, it is likely to trigger the slope instability phenomenon, which will ultimately lead to landslides and other geological disasters.

**Fig 21:**
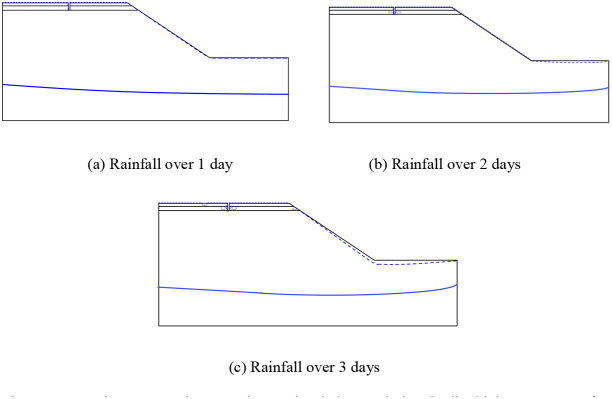
Transient saturated zone and water level changes in longitudinal joint pavements for different rainfall durations. (a) Rainfall over 1 day. (b) Rainfall over 2 days. (c) Rainfall over 3 days.

From Fig 21, it can be seen that: with the increase of rainfall time, the groundwater level rises continuously, and the transient saturated zone appears at the longitudinal joints of the road surface and the side slopes, and the transient saturated zone increases continuously with the increase of rainfall time. As can be seen from the figure, on the first day of rainfall, due to the small amount of rainfall, rainwater infiltration was only in the surface layer, so the demarcation line of the transient saturated zone appeared in the surface layer of the road surface and side slopes; on the second day of rainfall, due to the continuous infiltration of rainwater at the longitudinal joints, the longitudinal joints around the position of the location of the two droplet-shaped transient saturated zone, side slopes also appeared in the transient saturated zone of a smaller area; after three days of rainfall, due to continuous infiltration of rainwater, around the longitudinal joints and slopes, the transient saturated zone of the longitudinal joints and the slope also appeared. After 3 days of rainfall, due to the continuous infiltration of rainwater, the transient saturated zone around the longitudinal joint and at the slope becomes larger, and the transient saturated zone at the foot of the slope has a relatively larger extension range. According to the above analysis, it can be seen that the existence of longitudinal joints on the pavement transient saturated area and groundwater level changes have a certain impact. With the continuous increase in rainfall, the transient saturated area of the area will continue to grow, thus making the pavement substrate suction decreased, which ultimately will lead to the phenomenon of slope instability.

The following figure shows the transient saturated zone and the variation of groundwater level with rainfall time after polymer grouting the longitudinal joints of the pavement.

**Fig 22:**
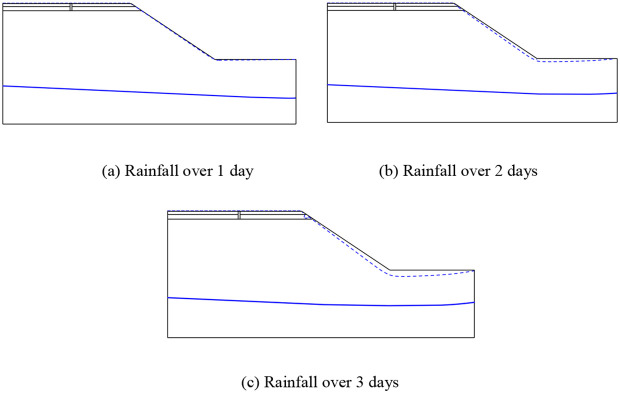
Transient saturated zone and water level changes in grouted pavement for different rainfall durations. (a) Rainfall over 1 day. (b) Rainfall over 2 days. (c) Rainfall over 3 days.

From Fig 22, it can be seen that with the increase of rainfall time, the longitudinal joint pavement repaired by grouting first appeared on the surface of the slope with a small transient saturated zone, the transient saturated zone under the action of continuous rainfall slowly became larger, and along the slope showed a trend of gradual decrease in the area from the bottom to the top. By comparing with Fig 3.7 the change of transient saturated zone of pavement containing longitudinal joints, it can be seen that the transient saturated zone at the longitudinal joints disappeared after the polymer grouting, and it mainly appeared at the side slopes. On the 3rd day of rainfall, it can be seen that a small transient saturated zone was generated at the rightmost edge of the subgrade, which was due to the large difference between the permeability coefficient of the subgrade and the soil base, resulting in the difficulty of rapid infiltration of rainwater into the lower layer of the soil base at this location, thus making the rainwater pile up and generate a transient saturated zone here. Groundwater level inside the pavement does not change significantly with the increase of rainfall time, mainly showing a small increase in the trend of change. In order to more intuitively analyse the transient saturated zone and the change of groundwater level after polymer grouting, the following is a comparison of the changes at the slope under different pavement conditions during three days of rainfall.

**Fig 23:**
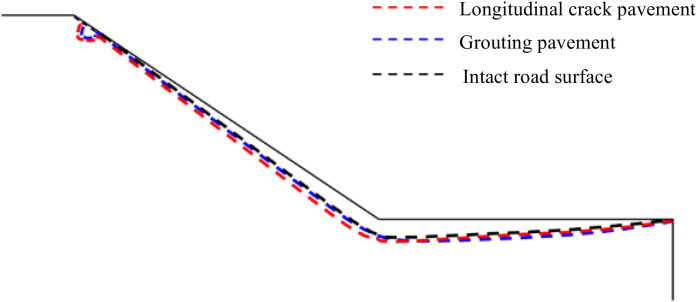
Distribution of transient saturated zones at 3 days of rainfall.

**Fig 24:**
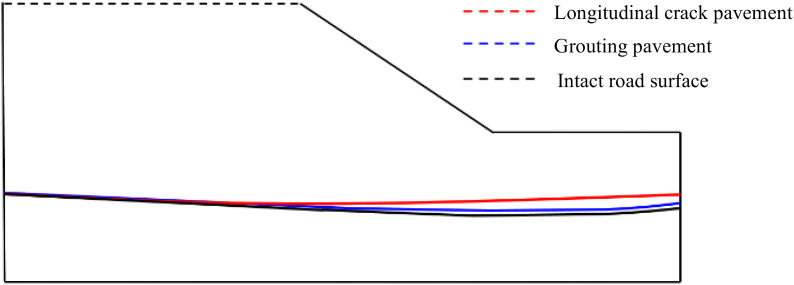
Distribution of groundwater levels at 3 days of rainfall.

From Fig 23, it can be seen that the area of transient saturated zone under the three conditions at the slope on the 3rd day of rainfall does not differ much, in which a small transient saturated zone appears at the edge of the base layer for both longitudinal joint pavement and grouted pavement, and the area of transient saturated zone at the foot of the slope of the grouted repaired pavement decreases to a certain extent compared with that of longitudinal joint pavement and is not very much different from that of the intact pavement. From Fig 24, it can be seen in the rainfall to the third day, containing longitudinal joint pavement groundwater level is higher than the grouted pavement higher than the intact pavement, and longitudinal joint pavement groundwater level height and the other two conditions of the largest difference. Through the above analysis, it can be concluded that after rainfall for a certain period of time, the effect of polymer grouting to repair longitudinal joint pavement is not obvious in the distribution of slope transient saturated zone, which is mainly reflected in the longitudinal joints at the repair and seepage characteristics of the improvement of the reason is mainly due to the grouting of the isolation of rainwater from the longitudinal joints of the infiltration so as to avoid the longitudinal joints at the production of transient saturated zone, and secondly, also makes it difficult for rainwater to seepage to the soil base through the longitudinal joints, which leads to the rise of groundwater level. Groundwater level rises, so the polymer grouting also shows a certain improvement and repair effect on the two seepage characteristics of transient saturated zone and groundwater level.

## 5. Conclusions

(1) In the pavement with longitudinal joints, rainwater will penetrate into the pavement along the longitudinal joints. With the increase of rainfall time, the pore water pressure at the longitudinal joints spreads downward slowly in the form of water droplets, and the maximum pore water pressure at the top of the soil base occurs close to the longitudinal joints and is close to 0. The magnitude of the change of the pore water pressure in longitudinal joints after grouting is relatively small and the maximum pore water pressure at the top of the soil base occurs in the side slopes, and the maximum pore water pressure close to longitudinal joints decreases by 46 2 kPa compared with that of pavements with longitudinal joints. The maximum pore pressure near the longitudinal joint decreased by 46.2 kPa compared with the pavement with longitudinal joint.
(2) When there is a longitudinal joint in the pavement, with the increase of rainfall time, the volumetric water content at the longitudinal joint and the slope changes at the fastest speed, the first to reach the saturation state, and the maximum volumetric water content at the top of the soil base appeared near the longitudinal joint, with the value of 0.185; the volumetric water content at the longitudinal joint with the change of rainfall is almost insignificant after the high-polymer grouting and the whole is close to that of the intact pavement. The maximum volumetric water content was 0.09, which was reduced by 0.095 compared with the maximum value of the pavement containing longitudinal joints.
(3) Compared with the longitudinal joint pavement during 3 days of rainfall, the transient saturated zone at the longitudinal joint disappeared after polymer grouting, and the area of transient saturated zone at the slope did not differ much in different working conditions, in which a small transient saturated zone appeared at the edge of the subgrade for both longitudinal joint pavements and grouted pavements, and the area of transient saturated zone at the foot of the slope of the grouted pavements decreased to a certain extent compared with the longitudinal joint pavement and the area of transient saturated zone at the foot of the slope of the grouted pavement decreased to a certain extent and did not differ much from that of the intact pavements. The difference is not significant. The groundwater level of the longitudinal jointed pavement is higher than that of the grouted pavement and higher than that of the intact pavement, and the change of the groundwater level of the grouted pavement is closer to that of the intact pavement.
